# Epigenetics of Stress, Addiction, and Resilience: Therapeutic Implications

**DOI:** 10.1007/s12035-014-9040-y

**Published:** 2014-12-11

**Authors:** Jean Lud Cadet

**Affiliations:** Molecular Neuropsychiatry Research Branch, DHHS/NIH/NIDA Intramural Research Program, National Institutes of Health, 251 Bayview Boulevard, Baltimore, MD 21224 USA

**Keywords:** Alcohol, Cocaine, DNA methylation, Heroin, Histone acetylation, Methamphetamine, Nicotine

## Abstract

Substance use disorders (SUDs) are highly prevalent. SUDs involve vicious cycles of binges followed by occasional periods of abstinence with recurrent relapses despite treatment and adverse medical and psychosocial consequences. There is convincing evidence that early and adult stressful life events are risks factors for the development of addiction and serve as cues that trigger relapses. Nevertheless, the fact that not all individuals who face traumatic events develop addiction to licit or illicit drugs suggests the existence of individual and/or familial resilient factors that protect these mentally healthy individuals. Here, I give a brief overview of the epigenetic bases of responses to stressful events and of epigenetic changes associated with the administration of drugs of abuse. I also discuss the psychobiology of resilience and alterations in epigenetic markers that have been observed in models of resilience. Finally, I suggest the possibility that treatment of addiction should involve cognitive and pharmacological approaches that enhance resilience in at risk individuals. Similar approaches should also be used with patients who have already succumbed to the nefarious effects of addictive substances.

## Introduction

Early stressful life events are important risk factors for the development of neuropsychiatric disorders that include affective disorders and addiction to food and illicit substances [1–7]. These traumatic events are associated with significant changes in cognitive, neurotransmitter, and neuroendocrine systems in humans and animal models [8–11]. Of interest is the fact that early stress events can cause significant changes in brain structure and function [12, 13]. Although stress-associated biochemical and structural alterations might constitute important subsets of general pathobiological substrates of psychiatric disorders, more experiments are needed to develop a theoretical framework that may have stronger translational impact on the lives of patients who suffer from substance use disorders (SUDs). SUDs are chronic neuropsychiatric disorders that are characterized by a compulsion to use licit or illicit substances, loss of control over drug use, and increased use despite adverse medical and psychological consequences [14, 15]. Some patients who are addicted to psychostimulants including cocaine and methamphetamine suffer from cognitive decrements that might impact their activities of daily living [16–18]. Given the fact that overwhelming stressful events can also be associated with some cognitive deficits [19, 20], it is not farfetched to suggest that repeated life events in addicted individuals might compound the adverse consequences of their illicit drugs of choice.

It is, nevertheless, important to note, at this juncture, that not all responses to stress are maladaptive since some of these responses might constitute resilient attempts to protect the individual against overwhelming odds [4, 13] that include living in deprived neighborhoods and parental deprivation [3, 21–23]. Indeed, evidence has accumulated to indicate that not all individuals develop maladaptive behaviors or psychiatric disorders despite living in dire conditions that include ethnic and social disparities [24–26]. In what follows, I will first try to draw comparisons between the epigenetic substrates of stress and addiction. I also provide a brief synopsis of epigenetic modifications observed in some models of resilience. Finally, I suggest that a better therapeutic handle of SUDs may be provided by promoting behaviors associated with resilience and by using, concomitantly, pharmacological interventions that trigger epigenetic changes identified in models of resilience.

## Brief Overview of Epigenetic Mechanisms

Epigenetics is defined as the investigation of heritable changes in gene transcription and/or phenotypic alterations that are not secondary to changes in DNA sequences [27]. This definition can be expanded to include meiotically and mitotically inherited alterations in gene expression that are not DNA-encoded [28–30]. The two most commonly studied epigenetic alterations are modifications of histones present in chromatin [31] and DNA methylation [32]. Chromatin represents the structural and functional organization of the eukaryotic genome [33, 34] and contains DNA, RNA, and several protein components [34]. The basic repeating unit of chromatin is the nucleosome that consists of 146 bp of DNA wrapped around four core histones, H2A, H2B, H3, and H4, which form an octomer (two of each core histone) [35]. Histone tails contain amino acid residues that can be reversely modified by histone acetyltransferases (HATs), histone deacetylases (HDACs), histone lysine methyltransferases (KMTs), and kinases, among others [36–38].

There are several classes of HATs, HDACs, and KMTs. The HAT families include cyclic AMP-responsive element binding (CREB)-binding protein (CBP)/p300, GNAT (general control non-repressible 5 (GCN5)-related N-acetyltransferases), and MYST named after its founder proteins [MOZ (also called MYST3, monocytic leukemia zinc-finger protein), YBF2, SAS2 (something about silencing 2), and TIP60 (60 kDa trans-acting regulatory protein of HIV-type 1 (tat)-interaction proteins)] subclasses [39, 40]. The GNAT HATs include GCN5 and CBP/p300-associated protein (PCAP). HDACs are subdivided into four classes according to sequence similarities [36]. These include Class I (HDAC1, HDAC2, HDAC3, HDAC8), Class II (HDAC4, HDAC5, HDAC6, HDAC7, HDAC9, HDAC10), Class III (sirtuins 1–7), and Class IV (HDAC11) HDACs [36, 41]. Class I, II, and VI HDACs are referred to as “classical” HDACs and are Zn^2+^-dependent enzymes [42], whereas the sirtuins require nicotinamide adenine dinucleotide (NAD)+ as a cofactor [43]. There are also several classes of KMTs that are involved in mono-, di-, and trimethylation of specific lysine residues on histones [44]. It needs to be kept in mind that methylation of histone H3K4 is generally associated with increased transcriptional activity [45] whereas methylation of H3K9 and H3K27 is associated with repression of gene expression [44, 46]. Moreover, several classes of lysine demethylases (KDMTs) counteract the effects of the KMTs [47]. Several HATs, HDACs, KMTs, and KDMTs are thought to play integral roles in the development of pathological states in both humans and animals [48, 49, 46].

DNA methylation and hydroxymethylation are represented by covalent modifications at the 5-position of cytosine to form 5-methylcytosine and 5-hydroxymethylcytosine, respectively [50–52]. DNA methylation is mediated by the de novo DNA methyltransferases, DNMT3A and DNMT3B, and by the maintenance methyltransferase, DNMT1 [52]. In contrast, ten-eleven translocation (TET1, TET2, and TET3) enzymes mediate active DNA demethylation [53]. These epigenetic enzymes are located in the brain and have been reported to play important roles in neurodevelopment, learning and memory, and in some neurologic and psychiatric disorders [54–57]. Several recent papers have also indicated a role for epigenetic modifications in the molecular processes that lead to addiction to psychostimulants including cocaine and methamphetamine [58–62]. These authors have also suggested that an approach that involves blocking the effects of the drugs on epigenetic markers might be beneficial to patients. In what follows, I suggest that any such approach will need to take into consideration environmental factors that might have influenced drug-induced epigenetic alterations in the brains of these patients.

## Epigenetic Bases of Responses to Stressful Events

Studies of environmental stress on epigenetic markers have documented unfavorable modifications that impact gene transcription in the brain and neuroendocrine systems [63–65]. Subsequent translational changes may in fact be the determining factors of the organism’s responses to both psychological and physical stresses [66, 67]. In the case of mild to moderate stresses, various species are able to cope by inducing general and/or stress-specific responses [68–70]. In the case of overwhelming stress, there is convincing evidence of significant stress-induced damage to the brain [13, 71]. During the past few years, several groups of investigators have reported that both acute and chronic stresses can impact the epigenome [72–75]. For example, a single immobilization stress alters hippocampal brain-derived neurotrophic factor (BDNF) gene expression and histone acetylation at BDNF gene promoters [73]. Berton et al. [76] also reported that chronic social defeat to repeated aggression caused increased BDNF in the brain. Importantly, BDNF knockdown in the nucleus accumbens (NAc) blocked the transcriptional effects of aggressive acts. Krishnan et al. [77] also showed that only mice sensitive to stress showed increased BDNF levels. Moreover, Roth et al. [78] have reported that a psychosocial stress regimen produced increased BDNF DNA methylation at exon IV and decreased mRNA expression in the dorsal hippocampus of adult rats.

Interestingly, hypersensitivity to stress was found to be due to loss of HDAC5, a class IIA HDAC [79]. In addition, chronic defeated mice show decreased expression of HDAC2, a member of class I HDACs, in the NAc [80]. There were also decreased levels of histone H3 acetylated at lysine 14 (H3K14Ac) in mice euthanized at 1 h after the final stress episode whereas there was increased H3K14Ac abundance at 24 h and 10 days after stress [80]. This study shows the time dependence of the effects of stress and withdrawal from stressful events. Mice susceptible to stress also show decreased expression of G9a (KMT1C) [81], an enzyme responsible for H3K9 methylation [82]. There was an associated decrease in the levels of H3K9 methylation in the NAc of these mice. A causal relationship between G9a and stress sensitivity was demonstrated by showing that increasing its expression in the NAc antagonized the effects of stress [82].

Other investigators have also assessed the role of epigenetic mechanisms in high responder (HR) and low responder (LR) rats [83]. HR rats show high locomotor activity while LR rats show low locomotor responses when exposed to a novel environment [84, 85]. The HR and LR dichotomy is a known predictor of behavioral and biochemical responses to addictive substances including cocaine and the amphetamines [86, 84, 87, 85]. The HR and LR rats were also reported to show differential epigenetic responses to stress, with HR rats exhibiting decreased H3K14 acetylation but the LR rats experiencing increased H3K14 acetylation [83]. These results indicate that these rats might show differential transcriptional responses to stress because acetylation of histones is an important regulator of gene expression [88]. It needs also to be pointed out that H3K14 acetylation is mediated, in part, by CBP [89], a histone acetyltransferase that plays an important role in the regulation of psychostimulant-induced behaviors and gene expression in the brain [90, 91].

In addition to stress encountered during adulthood, early life stresses can negatively impact the brain and behavioral outputs during both adolescence and adulthood [92]. These stresses include paternal and maternal deprivation [63, 93, 94]. Several investigators have also reported on the complex epigenetic effects of these types of stressors [95, 96]. In humans, prenatal exposure of maternal depression was associated with increased methylation of the neuron-specific glucocorticoid receptor (NR3C1) measured in genomic DNA obtained from cord blood of newborns [97]. Interestingly, McGowan et al. also reported decreased NR3C1 mRNA expression and increased DNA methylation at an NR3C1 promoter in the postmortem hippocampi of suicide victims who had a history of child abuse [98]. Moreover, Perroud et al. found changes in NR3C1 expression in adults with a history of child abuse, changes that were linked to the severity of the traumatic events [99]. These findings were further supported by the report that parental loss and childhood mistreatment were associated with increased NR3C1 promoter methylation in DNA obtained for leukocytes [100]. Interestingly, the adult rodent offsprings of high compared to low maternal care mothers show differential epigenetic changes in promoter regions and exons [95]. The epigenetic changes include decreased H3K9Ac enrichment, increased DNA methylation, and decreased expression of several genes in the low maternal care group [95]. Rats subjected to maternal deprivation for 2–13 days showed hypothalamic–pituitary–adrenal (HPA) axis hypersensitivity, increased corticotropin-releasing hormone (CRH) transcription in the paraventricular nucleus, and decreased DNA methylation at a CRE site in the CRH promoter [63]. More recently, convincing evidence has shown that offsprings of males that were subjected to post-traumatic stress showed impaired recognition memory, altered expression of genes involved in synaptic transmission and CREB phosphorylation pathways in the hippocampus, as well as abnormal hippocampal electrophysiological responses [101]. Therefore, when taken together, the accumulated literature indicates that early stressful events including maternal and paternal deprivation can cause long-lasting epigenetic changes that are measurable in adult mammals. It is, nevertheless, important to keep in mind that stressful events can cause epigenetically regulated biochemical, molecular, and structural changes in the brain at any period throughout an individual’s lifespan [102].

## Epigenetic Changes Associated with the Administration of Drugs of Abuse

Humans who suffer from SUDs constitute a group of individuals whose life spans are characterized by repeated stressful life events [103–105]. Indeed, traumatic events are risk factors for developing addiction to either licit or illicit drugs [2, 3]. These reports suggest that epigenetic alterations caused by stressful events might have rendered these individuals more susceptible to drug-induced neuroplastic changes that form the substrates of addictive diseases. In what follows, I provide a brief overview of some papers that have addressed the issue of drug-induced epigenetic changes in the mammalian brain. These drugs include, in alphabetical order, alcohol, cocaine, methamphetamine, nicotine, and opiates.

### Alcohol

The effects of alcohol on gene expression in the brain are well documented [106, 107]. More recently, several groups of investigators have begun to investigate potential epigenetic modifications that are secondary to alcohol exposure using various animal models [108, 109]. For example, Pandey et al. [110] had reported that an acute injection of alcohol caused decreased HDAC activity in the rat amygdala. They also found increased acetylation of histones H3 and H4 in the central (CeA) and medial (MeA) nuclei but not in the basolateral (BLA) nucleus of the amygdala. In contrast, alcohol withdrawal was associated with decreased histones H3 and H4 acetylation in the CeA and MeA. Moreover, alcohol withdrawal produced decreased expression of the HAT, CBP, in these brain amygdaloid nuclei. HDAC activity was, however, increased in the amygdala of similarly treated rats. D’Addario et al. [111] recently reported that binge administration of alcohol produced changes in the expression of prodynorphin and pronociceptin genes in the rat amygdala. The authors also reported increased acetylation of histone 3 at lysine 9 (H3K9Ac) but decreased abundance of H3 trimethylated at lysine 27 (H3K27me3) at the promoters of these two genes in animals treated with alcohol for 1 day. These histone modifications were also associated with increased prodynorphin and pronociceptin mRNA expression. Animals treated for 5 days showed only increased H3K9Ac at the pronociceptin promoter. Qiang et al. [112] also reported that withdrawal from chronic intermittent administration of alcohol increased H3K9Ac abundance at the glutamate receptor, NR2B. They also found decreased abundance of the methyltransferases, G9a and Suv39h1 (KMT1A), and of HDAC1-3 on the NR4B promoter region. Using a global chromatin immunoprecipitation (ChIP) technique, Zhou et al. [113] reported that there were significant changes in H3K4me3 abundance in a large number of genes in the hippocampus of alcoholics. However, these changes were not directly related to changes in the expression of any of these genes. A similar study also found marked changes in gene expression in the superior frontal cortex (CTX) as well as in the CeA and BLA of the amygdala [114]. Among these genes were mixed lineage leukemia (MLL also called KMT2A), MLL4 (KMT2B), and SET domain containing 1A (SETD1A also called KMT2F) that are involved in histone H3K4 trimethylation. The authors also observed increased H3K4me3 abundance at the promoters of BCL2L1 (B cell lymphoma 2-like1) and UBE1 (ubiquitin-like activating enzyme-1) whose mRNA levels are also increased in the brains of the alcoholic patients. In addition to alcohol-induced changes in histone markers, alterations in DNA methylation in the brains of alcohol abusing individuals have also been reported [115]. These authors reported that alcoholics showed higher methylation peaks than controls. Some of these genes of interest included HIST2H2AC and HIST1H4E, supporting the notion that alcohol abuse might be associated with altered histone modifications, as discussed above. In contrast, Ponomarev et al. [114] reported hypomethylation at DNA sequences called long terminal repeat (LTR)-containing retroposons. Taken together, these investigations identify multiple epigenetic alterations associated with alcohol administration.

### Cocaine

Cocaine causes substantial changes in gene expression in the brain [116–118]. However, the epigenetic bases of these transcriptional alterations needed to be clarified. Several groups of investigators have now published papers on the effects of cocaine on epigenetic markers in several brain regions [119]. These studies have included both acute and chronic effects of the drug. For example, Kumar et al. [120] reported that a single injection of cocaine increased c-fos mRNA levels and increased histone H4 acetylation at the c-fos promoter. In contrast, chronic cocaine did not induce c-fos mRNA nor histone hyperacetylation. Moreover, the authors detected increased histone H3 acetylation at the BDNF promoter and increased BDNF mRNA levels. Subsequently, Levine et al. [90] documented a role for the acetyltransferases, CBP, which was found to control the effects of cocaine via acetylation of histones at the fosB promoter. Malvaez et al. [91] have also identified a role for CBP and histone acetylation in cocaine-induced behaviors. Moreover, Romieu et al. [121] showed that HDAC inhibitors could decrease cocaine self-administration, documenting roles for histone acetylation in cocaine-induced behaviors and molecular effects. This suggestion is supported by the report that cocaine self-administration caused increased HDAC2 and HDAC11 expression [122]. Cocaine self-administration also caused decreased HDAC5 accumulation in the nucleus, suggesting that this class IIA HDAC may, in part, regulate some of cocaine-induced molecular effects in the brain. In addition to HDAC5 [122, 123], other HDACs including HDAC1 [124], HDAC3 [125], and HDAC4 [126] have also been implicated in cocaine-induced behavioral changes. The class III HDACs including SIRT1 and SIRT2 also participate in the actions of cocaine in the brain [127, 128].

Other investigators have also documented long-term changes in gene expression after cocaine self-administration, with some of these changes being related to differential alterations in histone H3 acetylation [118]. One gene that is upregulated by chronic cocaine is CaMKIIalpha [126]. CaMKIIalpha is a very important kinase in the adult brain and plays important roles in synaptic plasticity and in mechanisms involved in learning and memory [129]. It is also of interest that increased BDNF expression observed after cocaine withdrawal [130] also involves increased histone acetylation at the BDNF exon-I promoter [131]. Importantly, some of the epigenetic effects of cocaine are dependent on stimulation of DA D1-dependent signaling pathways [132]. In addition to histone acetylation, cocaine-induced neuroadaptations appear to be also dependent on histone methylation because repeated exposure to cocaine produced decreased global levels of H3K9me2 and decreased expression of the methyltransferase, G9a, in the nucleus accumbens [133]. Interestingly, repeated injections of THC, the active ingredient of marijuana, also caused decreased H3K9me2 but increased H3K4me3 at sites flanking the proenkephalin transcription start site (TSS) using tissues from the shell subdivision of the rat NAc [134]. Cocaine also increased the expression of methyl-CpG binding protein 2 (MeCP2) and produced de novo DNA methylation [122, 135]. Moreover, exposure to cocaine increased MeCP2 phosphorylation [136, 137]. Importantly, knockdown of MeCP2 in the dorsal striatum was shown to decrease cocaine intake by regulating BDNF levels in that structure [138]. Furthermore, Anier et al. [139] reported that acute cocaine caused upregulation of DNMT3A and DNMT3B in the mouse NAc. Cocaine also caused hypermethylation and increased MeCP2 binding at the promoter of the protein phosphatase-1 catalytic subunit (PP1c) and decreased PP1c mRNA expression. The reverse was true for the effects of cocaine on the fosB promoter and fosB mRNA levels. Together, these findings suggest that cocaine can trigger epigenetic alterations that might influence, in the long term, memories associated with cocaine-related behaviors.

### Methamphetamine

Although methamphetamine is a highly addictive drug with a higher prevalence than cocaine abuse throughout the world, basic mechanisms associated with methamphetamine addiction have been less well studied than those of cocaine. In the past, many studies have been dedicated to toxic effects caused, in part, by allostatic load due to drug-induced release of high levels of dopamine in the dorsal striatum [140]. Some studies have also focused on behavioral models such as self-administration [141] and the effects of these drugs on gene expression [142, 143]. More recently, a few groups of investigators have begun to carry experiments to elucidate potential epigenetic effects of this drug [58]. Specifically, Martin et al. [144] reported that an acute injection of methamphetamine caused decreased abundance of histone H3 acetylated at lysine 9 (H3K9Ac) and at lysine 18 (H3K18Ac) in the rat NAc. There was also methamphetamine-induced hyperacetylation of H4K5 and H4K8. The increased H4 acetylation is consistent with the results of Harkness et al. [145] who also reported that acute methamphetamine also increased H4 acetylation in the striatum. These results are also consistent with the report that acute methamphetamine increased the expression of several immediate early genes and that these increases were correlated with methamphetamine-induced increased binding of acetylated H4K5 on the promoters of these genes [146]. The increased histone acetylation may be the result of drug-mediated decreased HDAC1 expression since the methamphetamine injection produced decreased HDAC1 expression in nuclear sub-fractions from the NAc [144]. The possibility that the methamphetamine-induced increased H4 acetylation might be due to increase in CBP expression also needs to be evaluated because increased CBP expression is associated with increased histone acetylation [147]. This possibility is bolstered by the fact that methamphetamine self-administration can regulate gene expression via CREB phosphorylation [60]. In any case, the results of the acute methamphetamine injection suggest that both HDAC1 and HDAC2 might participate in the regulation of methamphetamine-induced changes in gene expression in the brain. In the NAc, acute methamphetamine injection also causes increased expression of the mitochondrial sirtuins, SIRT3 and SIRT5, sirtuins known to play an integral part in regulating mitochondrial functions [148, 149]. Other studies conducted by Jayanthi et al. [59] have also documented that chronic methamphetamine can cause increased expression of HDAC1, HDAC2, SIRT1, and SIRT2 in the dorsal striatum. Those authors also reported increased expression of other epigenetic proteins including MeCP2, REST, and Co-REST that are members of co-repressor complexes with class I HDACs that serve to regulate gene transcription [150–152]. Methylation of histone H3 at lysine 4 (H3K4 me2/3) also appears to be important in methamphetamine-induced conditioned place preference (CPP) because increased methylation is associated with increased CPP whereas decreased methylation is related to decreased CPP [153].

Related to the above discussion on stress is the fact that maternal separation was found to promote greater methamphetamine self-administration and to enhance the effects of the drug on MeCP2 expression in the core of the nucleus accumbens [154]. These results are consistent with the possibility that methamphetamine might produce increased DNA methylation because the drug increases DNMT1 expression in the brain [59, 155]. Indeed, it was recently reported that mice that had been exposed to methamphetamine in utero showed differentially DNA methylation in their hippocampi [156]. When taken together, these results indicate that methamphetamine can also exert a multiplicity of epigenetic changes in the brain.

### Nicotine

The effects of nicotine on gene expression in the brain have been documented [157, 158]. Recently, there has also been a focus on identifying potential nicotine-induced epigenetic alterations [159]. For example, chronic nicotine has been reported to enhance cocaine-induced synaptic plasticity by increasing H3K9 and total H4 protein acetylation that was associated with increased H3K9Ac and total H4Ac binding to the FosB promoter in samples obtained from the mouse ventral striatum [160]. These nicotine-induced changes in histone acetylation were secondary to decreased HDAC activity and accompanied by increased FosB mRNA expression. Interestingly, use of the HDAC inhibitor, suberoyl ailide hydroxamine acid (SAHA), was reported to mimic the effects of nicotine on the physiological effects of cocaine. A subsequent study by the same group also found that nicotine could also enhance cocaine-induced physiological changes via HDAC inhibition [161]. Another group of investigators reported that repeated subcutaneous injections of nicotine produced increased expression of dopamine D1 receptors in the rat frontal cortex [162]. These increases were associated with increased H4 acetylation at the D1 receptor promoter. In addition to increased histone acetylation, others have shown that exposure to nicotine can decrease the expression of histone methyltransferases including G9a and Setb1in the mouse cortex [163]. This pattern of nicotine administration also increased BDNF expression that was mediated, in part, by decreased H3K9me2 binding to the BDNF promoter. Nicotine injections also decreased the mRNA and protein expression levels of the DNA methylation enzyme, DNMT1, in the mouse cortex and hippocampus [164]. Nicotine also produced increased cortical GAD67 mRNA expression that was accompanied by decreased levels of GAD67 promoter methylation. Together, these papers support the notion that nicotine can produce changes in gene expression via diverse epigenetic alterations.

### Opiates

Administration of heroin and other opiates alters the expression of genes involved in multiple molecular pathways [116, 165, 166]. A few studies have now been conducted on the role of epigenetic mechanisms in opiate-mediated behaviors. For example, heroin CPP produced dose-dependent histone H3 phosphoacetylation in the nucleus accumbens [167]. Morphine context-associated memory is enhanced by injections of the HDAC inhibitor, trichostatin A (TSA), directly into the BLA of the amygdala [168]. These injections led to increased H3K14 acetylation and increased BNDF expression. Other investigators have demonstrated that morphine withdrawal is associated with decreased histone H3K9 trimethylation at BDNF promoters II and III in the VTA and locus coeruleus (LC) but increased H3K9/14 acetylation at the BDNF promoter II only in the LC [169]. These epigenetic modifications were consistent with increased BDNF mRNA levels in morphine-withdrawn rats. Another interesting study reported that chronic morphine administration did not cause any changes in histone H3 phosphorylation [170]. However, naltrexone-induced withdrawal produced increased H3 phosphorylation that was mediated, in part, by ERK-dependent mechanisms in the rat NAc and the lateral septum. There was also increased H3K14 acetylation in the shell of the NAc. Chronic morphine decreased G9a expression and global levels of H3K9me2 in the mouse NAc [171]. G9a upregulation in the NAc also interferes with morphine CPP and locomotor sensitization. In comparison to control mice, morphine also caused marked changes in global H3Kme2 binding in the mouse NAc, as detected by ChIP-Seq, with 8103 sites being downregulated but 5669 being upregulated. Three glutamate receptor genes (Grin2A, Grm5, and Grm8) that showed decreased H3K9me2 binding also showed increased mRNA levels, thus implicating glutamatergic systems in opiate addiction [171]. The effects of opiates on DNA methylation have also being investigated, without there being any significant changes observed in the mouse brain after chronic intermittent heroin administration [172].

In summary, this overview of the molecular effects of various drugs of abuse suggests that these substances can produce a multitude of epigenetic modifications whether animals were injected by experimenters or were put through a self-administration paradigm. Nevertheless, the specific role of these epigenetic alterations in the development of truly addicted states remains to be elucidated further.

## Psychobiology of Resilience

Resilience refers to a relative protection of an individual or family against environmental stresses to which others might be prone to succumb [173–176]. Levels of resilience have been shown to predict hopelessness that is a harbinger of future affective disorders [177, 178]. Resilience may also explain the fact that not all adolescents or adults who live in areas of deprivation based on ethnic and socioeconomic factors become addicted to either licit or illicit drugs [179–183]. There also appear to be gender differences in resilient outcomes, with women showing more resilience than men [184]. In humans, interactions between family characteristics, community involvement, and genetic markers may confer sensitivity to increased morbidity to a number of medical and psychiatric illnesses including SUDs [179, 180, 185]. Some of the genetic markers include polymorphism in genes that encode dopamine receptors and the serotonin transporter [179, 180, 185], with socioeconomic status influencing the trajectory of pathologies associated with these polymorphisms [186].

In addition to potential individual genetic predilections [187], evidence has been collected in animal models that epigenetic modifications might also play a role in the development of resilient phenotypes [81, 188]. As mentioned above, resilient mice that were exposed to chronic stress do not show abnormalities in the expression of the G9a histone methyltransferase enzyme in their NAc whereas susceptible animals do [81]. Stressed animals that were susceptible to stressful events showed differential abundance of histone H3K27 methylation at several genes whereas resilient mice showed patterns that were similar to normal animals [189]. Uchida et al. [190] also published an interesting study showing that stress can produce epigenetic regulation of striatal GDNF (glial cell-derived neurotrophic factor) responses in mice that differ in their susceptibility to stress.

There is also evidence that coping/resilient mechanisms in response to stressful stimuli can be enhanced in various ways. For example, the accumulated evidence suggests that the HPA response to stressful events is influenced by maternal behaviors [191, 192]. In rodents, these responses are influenced by maternal care and are mediated by epigenetically determined changes in gene expression [193–195]. These maternal behavior-induced epigenetic changes include differences in DNA methylation and histone acetylation and binding of the transcription factor, NGFIA (nerve growth factor 1A), at the promoter of the glucocorticoid receptor [194], with some degree of reversibility through a dietary manipulation [196]. Of singular importance to the theme being promulgated here is the fact that these maternal behaviors can also influence future daughters’ behaviors when they, themselves, become mothers [197, 198]. Moreover, Gonzalez et al. [199] had reported that maternal deprivation could have negative intergenerational effects on the behaviors of female rats, with other investigators reporting similar findings [200]. Furthermore, Shoji and Kato [201] have investigated the development of maternal behaviors in BALC/c and CBA/Ca mice that differ in parenting behaviors, with the CBA/Ca being better mothers. The authors demonstrated that cross-fostering of BALB/c pups by CBA/Ca mothers improves the future mothering behaviors of BALB/c females that show, when they become mothers, increased licking and grooming of their own pups. The evidence reviewed here suggests that training human mothers to provide good maternal care may have positive trans-generational effects within communities affected by socioeconomic adversities. Similar arguments can be put forward for paternal care [202, 203]. Mychasiuk et al. [202] also reported that paternal stress had negative impact on behaviors and increased DNA methylation in the hippocampus of their offsprings. In contrast, enrichment of the environment of male Long Evans rats with toys, multiple levels of exploration, and several cage mates for 28 days before mating with control female rats had positive impact on exploratory behaviors of the males’ offsprings and on DNA methylation in their hippocampi and frontal cortices [203]. Enrichment of paternal environment can also have positive effects on maternal care and pup behaviors [204]. Although more research is needed to investigate the potential effects of these kinds of manipulations on future drug taking in animal models, the reviewed observations suggest that promoting resilience may impact drug self-administration in the offsprings of parents reared in enriched environments.

## Therapeutic Implications: Promoting Resilience Against Substance Use Disorders

In the past two decades, there has been a heavy reliance on the potential of brain science to explain the cause, development, and clinical course of SUDs [205]. This reductionist construct has led to a large number of important basic science discoveries that have not yet significantly impacted the daily lives of patients who suffer from these recalcitrant disorders. This statement is not only true for SUDs but also for several medical and psychiatric disorders where there are obvious disparities based on ethnic and socioeconomic status [206–208]. In the case of SUDs, the almost complete theoretical reliance on the behavioral, biochemical, and epigenetic observations in animals to explain human addiction might have corrupted our efforts to develop therapeutic approaches. This is because the focus has almost solely been on potential pharmacological “magic bullets” for a quick fix of the addictions. Similar approaches to other complex neuropsychiatric illnesses have not necessarily met with greater therapeutic outcomes. In fact, this reductionist emphasis might have led to a closure of our minds to the potential for families, communities, and other sources of enrichment to enhance resilience in individuals who are at risk [209] or are already suffering from SUDs.

As stated above, not everybody subjected to gross disparities ends up with a diagnosis of SUDs. This fact implicates individual as well as family- and community-based resilient factors in protecting these individuals against drug addiction [180, 210, 211]. These observations notwithstanding, instead of pursuing an agenda that actively promotes enhancement of resilience by reinforcing identified protective factors [212–214], the focus has been mainly on identifying negative or pathological elements in these communities [215]. I suggest that a more profitable approach for our addicted patients may be to identify resilient factors such as coping styles because these characteristics have been shown to reduce vulnerability to medical and psychiatric disorders in humans [216, 217]. There is also evidence, in animal models, that promotion of active coping can have significant neuroendocrine and epigenetic effects [218] whereas maternal deprivation can influence drug-taking behaviors [94]. In fact, an agenda that tries to identify resilient factors would have the added benefit of improving the treatment of patients who are co-morbid for SUDs and other psychiatric disorders [219].

This discussion further suggests a need for a research emphasis that attempts to identify coping styles of individuals and families that show resilience against the ravages of SUDs although they are still living in environments marred by poverty, poor access to education, or repeated discriminatory aggressive acts [220]. Once these coping styles are identified, it may be possible to enhance these behavioral patterns through active mentoring of individuals at risks, in effect, recognizing the potential prominence of social interactions in the development of phenotypic diversity within specific environments. Because the effects of life stresses [221] and resilient behaviors [203] can be transmitted across generations, these environmental interventions may have substantial positive cost/benefit ratios for our health care systems and our nations by negating the complex effects of environmental stresses on the behaviors of young children and adolescents reared in these settings. This conclusion is supported by the high co-morbidity of other psychiatric diatheses and SUDs and the fact that some of these disorders appear to share similar developmental risks and potential epigenetic substrates [222, 223].

**Fig. 1:**
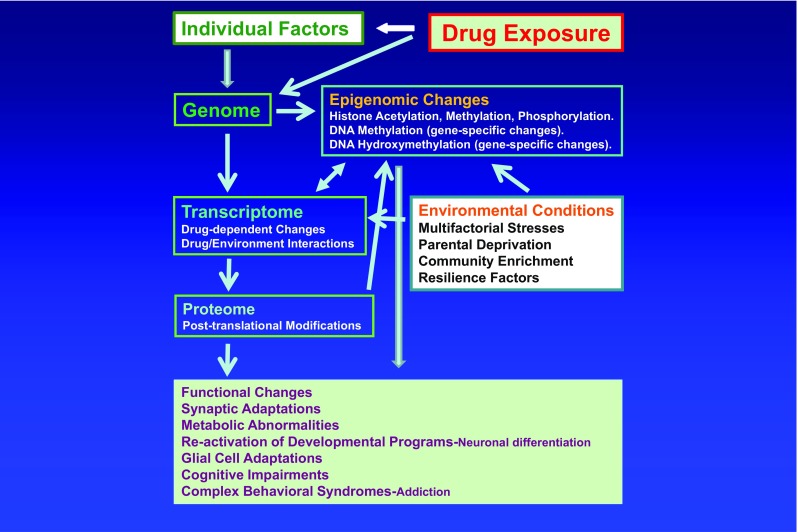
Schema showing the potential interactions of drugs of abuse with an individual’s genome and the impact of environmental vicissitudes on the individual’s responses to these agents. The epigenetic responses to the drug will probably be dependent on the genetic background, family resilient factors, and environmental stressors that individuals face during their lifetimes. Substance use disorders (addiction) are thus viewed not as reductionist constructs but as multifactorial complex neuropsychiatric disorders, with only a few individuals actually developing those syndromes after trying various rewarding substances. This statement suggests the need to develop more animal models that take these issues into consideration. By extension, our pharmacological treatments may thus be bound to fail because present approaches of developing therapeutic agents employ all animals that self-administer a drug in question whereas only a few percentages of humans become addicted to a licit or illicit substance. The schema also suggests the need to identify resilient factors within individuals and families that treatment personnel can shore up within addicted individuals and teach to those who are living in high-risk situations

This thesis does not negate the need for continued support of reductionist approaches that provide useful neurobiological explanations for the direct effects of drugs on the brain. It hints, however, to the added possibility of using therapeutic agents such as antidepressant drugs alone or in combination with other epigenetic agents that have been shown to promote resilience in animal models [81]. I argue, however, that these pharmacological agents may be more beneficial when used in conjunction with mentoring activities that promote active coping. This proposal also points to the need to develop better animal models that are more representative of human conditions [224, 225, 141, 226] since not everyone who experiments with drugs is or will become a drug addict. The development of better models will help to differentiate biochemical and epigenetic effects observed after simple drug exposure from those alterations associated with true addicted states. The need to use better animal models of addiction in molecular studies is illustrated well by recent papers that have purported to identify nicotine-induced epigenetic modifications that were proposed as supporting evidence for the idea of nicotine as a gateway agent to psychostimulant abuse [159]. I argue further that these new models would need to go beyond superficial similarities between animal responses to drugs of abuse and the complex cognitive behaviors and affective states observed in our addicted patients. As a consequence of using better models, pharmacologic agents derived from these experiments would be more specific in terms of blocking or suppressing epigenetic, transcriptional, and biochemical changes associated with sensitivity to stress and SUDs. By extension, these medications may have a greater impact on the lives of our patients. Using this line of reasoning, SUDs will need to be viewed not as being secondary solely to drug-induced biochemical and epigenetic effects but as, most likely, to be secondary to interactions of drugs with the genomes of individuals living within an environment that was permissive to the development of pathologic use of drugs (see Fig. 1 for a schematic rendering). This statement re-emphasizes the need to always bear in mind the existence of resilient factors or traits in individuals, families, and sub-communities that have shown their utilities in combating the effects of drugs in individuals, even when said individuals had experimented with addictive substances during their adolescence. I believe that this capacity to thrive against inordinate odds can be strengthened through mentored good mothering.

## Abbreviations

BDNF: Brain-derived neurotrophic factor
BLA: Basolateral nucleus of the amygdala
CREB: Cyclic AMP-responsive element-binding protein
CBP: CREB-binding protein
CeA: Central nucleus of the amygdala
CPP: Conditioned place preference
CREB: Cyclic AMP response element binding
DNMT: DNA methyltransferases
GDNF: Glial cell-derived neurotrophic factor
GCN5: General control non-repressible 5
GNAT: GCN5-related N-acetyltransferase
H3K14Ac: Histone H3 acetylated at lysine 14
H3K9Ac: Histone H3 acetylated at lysine 9
H3K18Ac: Histone H3 acetylated at lysine 18
H3K4me2/3: Methylation of histone H3 at lysine 4
HATs: Histone acetyltransferases
HDACs: Histone deacetylases
HPA: Hypothalamic–pituitary–adrenal
HR: High responders
LC: Locus coeruleus
LR: Low responders
KMTs: Histone lysine methyltransferases
KDMTs: Lysine demethylases
MeA: Medial nucleus of the amygdala
MeCP2: Methyl-CpG binding protein 2
MYST: MOZ YBF2, SAS2, and TIP60
NAc: Nucleus accumbens
NAD: Nicotinamide adenine dinucleotide
NGF1A: Nerve growth factor 1A
NR3C1: Neuron-specific glucocorticoid receptor
PCAP: p300 CBP/p300-associated protein
SIRT: Sirtuin
SUDs: Substance use disorders
TET: Ten-eleven translocation
